# The multiscale nexus among land use-land cover changes and water quality in the Suquía River Basin, a semi-arid region of Argentina

**DOI:** 10.1038/s41598-024-53604-0

**Published:** 2024-02-26

**Authors:** Sofía Paná, M. Victoria Marinelli, Matías Bonansea, Anabella Ferral, Donatella Valente, Vera Camacho Valdez, Irene Petrosillo

**Affiliations:** 1 Consejo Nacional de Investigaciones Científicas y Técnicas (CONICET), Av. Cdad. de Valparaíso S/N, Córdoba, Argentina; 2 Instituto Gulich, Centro Espacial Teófilo Tabanera, Universidad Nacional de Córdoba-CONAE, Ruta 45 km 8, Falda del Cañete, 5187 Córdoba, Argentina; 3 Departamento de Estudios Básicos y Agropecuarios, Facultad de Agronomía y Veterinaria (FAyV), Universidad Nacional de Río Cuarto (UNRC), Río Cuarto, Argentina; 4 Laboratory of Landscape Ecology, Department of Biological and Environmental Sciences and Technologies, University of Salento, Prov. Le Lecce-Monteroni, 73100 Lecce, Italy; 5 CONAHCYT- Departamento de Conservación de la Biodiversidad, El Colegio de la Frontera Sur, San Cristóbal de las Casas, México

**Keywords:** Ecology, Environmental chemistry

## Abstract

Agricultural intensification and urban sprawl have led to significant alterations in riverscapes, and one of the critical consequences is the deterioration of water quality with significant implications for public health. Therefore, the objectives of this study were the assessment of the water quality of the Suquía River, the assessment of LULC change at different spatial scales, and the analysis of the potential seasonal correlation among LULC change and Water Quality Index (WQI). The Sample Sites (SS) 1 and 2 before Cordoba city had the highest WQI values while from SS3 the WQI decreased, with the lowest WQI close to the wastewater treatment plant (SS7) after Cordoba city. From SS8 in a agricultural context, the WQI increases but does not reach the original values. In light of analysis carried out, the correlation between water quality variables and the different LULC classes at the local and regional scales demonstrated that WQI is negatively affected by agricultural and urban activities, while natural classes impacted positively. The spatialization of the results can help strongly in assessing and managing the diffusion of point and non-point pollution along the riverscape. The knowledge gained from this research can play a crucial role in water resources management, which supports the provision of river ecosystem services essential for the well-being of local populations.

## Introduction

Riverscapes play a crucial role in supporting and providing diverse ecosystem services such as biodiversity conservation, climate regulation, carbon sequestration, groundwater recharge, and water supply^1,2^. However, climate change and human activities, particularly agricultural intensification, urban sprawl, and urban stormwater runoff during precipitation events have led to significant alterations in these ecosystems^3–5^. These alterations pose a threat to environmental quality and the ability of riverscapes to provide essential ecosystem services, which are vital for the well-being of nearby communities^6–8^.

One of the critical consequences of human-induced changes in riverscapes is the decline of water quality, which is recognized as a global concern with significant implications for public health^9–11^. Taking into account this challenge, the Agenda 2030 for Sustainable Development Goals focuses on addressing the impact of anthropogenic activities on water quality^12^.

Land use and land cover (LULC) changes can alter the hydrologic patterns of different landscapes, contributing to the proliferation of non-point sources of pollution, ultimately affecting the quality of water resources^13–17^. Given that LULC changes can affect the river ecosystems, the availability of comprehensive data on LULC patterns and their temporal changes is crucial for effectively managing variations in water resource quality and quantity. In this perspective, to effectively study the relationship between water quality and LULC change, remote sensing and satellite information have emerged as valuable tools^18–20^. In particular, remote sensing data offer several advantages over alternative approaches for obtaining such data^21–24^: (1) they provide access to multispectral and multitemporal open source data; (2) these data sources offer global coverage and several spatial information and resolutions; and (3) assessments based on satellite data can enhance the understanding and control of water resource pollution, providing guidelines for effective watershed management, and land use planning that prioritize the provision of basic ecosystem services to local populations. This LULC change-water quality nexus must be addressed in a multi-scale approach, considering multiple buffer zones^25,26^. According to Kim et al.^27^, since the buffer size could affect the accuracy of the results, it must be customized to match the study objectives and the watershed characteristics.

In the past, Pesce and Wunderling^28^ developed a Water Quality Index (WQI) to assess the spatio-temporal dynamics of water quality in the Suquía River (Argentina), highlighting that Córdoba City produced a severe negative effect on water quality. More recently, Zambrano et al.^29^ have related the WQI developed by Pesce and Wunderlin^28^ to pesticide concentrations in the Suquía River and have correlated the negative impact of water quality degradation with reproductive organ abnormalities in the native fish species *Cnesterodon decemmaculatus*. In the case of the Suquía River, some authors have reported high levels of inorganic and organic pollution related to point and non-point sources^29–34^: hormones, antibiotics, fecal coliforms, and viruses have been found into this river, clearly indicating a severe anthropogenic impact on water quality, which threatens aquatic biodiversity and human health. However, few studies have been focused on the effects of LULC change on water quality variables using field data, remote sensing techniques, and statistical approaches^35,36^. In this sense, LULC change can play a crucial role since Suquía River flows in a riverscape characterized by agricultural intensification of some crops of global economic interest such as soybeans and corn^37^.

Since rivers are affected by the human activities carried out in their riverscape^38,39^, the aims of this research are (1) the assessment of the water quality of the Suquía River for the 2018–2019 period by implementing the WQI developed by Pesce and Wunderling^28^; (2) the assessment of LULC change for the same period to analyze the riverscape dynamics at different spatial scales, and (3) the spatial analysis of the relation between LULC change, water quality parameters, and WQI by taking into consideration the seasonal variability. In this perspective, this research presents a novel spatial approach by integrating WQI and LULC dynamics. It can implement the knowledge gap interesting the Suquía River but it can represent a reference approach to be applied in a riverscape where different drivers can act simultaneously under seasonal changing conditions, thus providing useful indicators worldwide.

## Methods

### Study area

**Figure 1:**
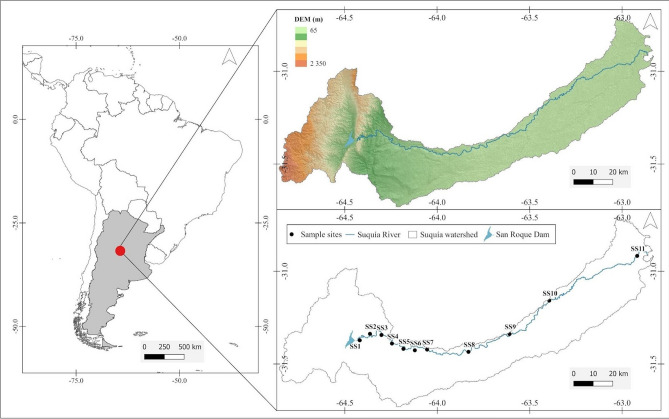
Geographical location of Suquía River Basin and sampling sites along the river.

The Suquía River Basin is located in a semi-arid argentinian region, where rainfall is in the range of 700–900 mm per year, mainly concentrated from October to March/April^40,41^. The basin covers an area of 7700 km^2^. The Suquía River flows for approximately 200 km, predominantly in a west-northeast direction, from the San Roque dam to the Mar Chiquita Lagoon in northeastern Córdoba Province. The headwaters of the basin are located in a mountainous region, and the middle and lower basins are characterized by the development of agricultural and livestock activities (Fig. 1). The average flow of this river during the rainy season, from December to April, is estimated to be greater than 15 m^3^ s^−1^. In contrast, in the dry season, from May to November, it is estimated to be less than 10 m^3^ s^−1^, with a minimum value in June (5/6 m^3^ s^−1^)^28,42^. The Suquía River flows through several cities, including Córdoba city, the second largest and most populated city in Argentina with more than 1.5 million inhabitants accroding to the Instituto Nacional de Estadísticas y Censos (2022), where the Suquía River is the main source of water for human consumption.

### Water quality assessment

To assess the water quality of the Suquía River, a dataset provided by the Provincial Administration of Water Resources of Córdoba Province (APRHI) was used, which contains water quality field data from 2018 to 2019 collected in eleven sampling sites (SSs) along the river (Fig. 1). A Garmin GPS model eTrex Vista HCx was used to reach monitoring points with an accuracy of 15 m or less. In situ measurements as pH, dissolved oxygen, water temperature, and conductivity were registered with a Horiba U-22 multiparametric probe in each site, at 0.2 m depth and water samples were taken through manual point collection at the same depth. Sampling, storage, preservation, and analysis of water samples were carried out in the laboratory according to the American Public Health Association (APHA) methodology proposed by the Standard Methods for Water and Wastewater (SMWW)^43^. The dataset included the following variables: temperature (SMWW-Met-2550 Temperature B), pH (SMWW-Met-4500-H + B), dissolved oxygen (SMWW-Met-4500-O G), color, conductivity (SMWW-Met-2510 B), turbidity (SMWW-Met-2510 B), total evaporation residue (SMWW-Met-2540 A, total alkalinity (SMWW-Met-2510 B), chloride (SMWW-Met-4500-Cl B), hardness (SMWW-Met-2340 B), sulfate (SMWW-Met-4500 E), total phosphorus (SMWW-Met-4500-P A-E), ammonium (SMWW-Met-4500 Met-NH3), nitrite (SMWW-Met-4500 NO2 B), nitrate (SMWW-Met-4500 NO3 E), and total coliform bacteria (SMWW-Met-9221 E). Sampling campaigns were taken at a seasonal frequency, representing the dry and cold prevalent climatic conditions of winter, and the wet and warm prevalent climatic conditions of summer.

#### Water Quality Index

The WQI developed by Pesce and Wunderling^28^ was employed to assess the water quality of the Suquía River in each sample site. Considering the different climatic seasons three WQI values were calculated: an overall average WQI and two separate WQIs corresponding to the summer and winter conditions. Equation (1) presents the WQI formula, while Table 1 provides the coefficients and weights assigned to each water quality variable used in the calculation of the WQI.

1 $$ WQI = \frac{{\sum\nolimits_{i} {C_{i} xP_{i} } }}{{\sum\nolimits_{i} {P_{i} } }} $$

where C_i_ represents the value assigned to each parameter (*x*) after normalization, and Pi is the relative weight assigned to each parameter.

**Table 1:** Normalization factor (C_i_) and relative weight for parameters considered to calculate the Water Quality Index^28^.

Parameter	Relative weight (Pi)	Normalization factor (C_i_)										
		100	90	80	70	60	50	40	30	20	10	0
Conductivity	2	< 750	< 1000	< 1250	< 1500	< 2000	< 2500	< 3000	< 5000	< 8000	≤ 12,000	> 12,000
Dissolved oxygen	4	≥ 7.5	> 7	> 6.5	> 6	> 5	> 4	> 3.5	> 3	> 2.0	≥ 1.0	< 1.0
Turbidity	2	< 5	< 10	< 15	< 20	< 25	< 30	< 40	< 60	< 80	≤ 100	> 100
Nitrite	2	< 0.005	< 0.01	< 0.03	< 0.05	< 0.10	< 0.15	< 0.20	< 0.25	< 0.50	1.00	> 1.00
Nitrate	2	< 0.5	< 2.0	< 4.0	< 6.0	< 8.0	< 10.0	< 15.0	< 20.0	< 50.0	100.0	> 100.0
Ammonium	3	< 0.01	< 0.05	< 0.10	< 0.20	< 0.30	< 0.40	< 0.50	< 0.75	< 1.00	1.25	> 1.25
Hardness	1	< 25	< 100	< 200	< 300	< 400	< 500	< 600	< 800	< 1000	1500	> 1500
Chloride	1	< 25	< 50	< 100	< 150	< 200	< 300	< 500	< 700	< 1000	1500	> 1500
Sulfate	2	< 25	< 50	< 75	< 100	< 150	< 250	< 400	< 600	< 1000	1500	> 1500
Total coliform bacteria	3	< 50	< 500	< 1000	< 2000	< 3000	< 4000	< 5000	< 7000	< 10,000	14,000	> 14,000
Temperature	1	21/16	22/15	24/14	26/12	28/10	30/5	32/0	36/ − 2	40/ − 4	45/ − 6	> 45/ < − 6
pH	1	7	7–8	7–8.5	7–9	6.5–7	6–9.5	5–10	4–11	3–12	2–13	1

### Satellite LULC maps

To analyze the LULC change during the 2018–2019 period, the LULC thematic maps were elaborated through the map fusion technique performed with ENVI, and the final maps were obtained using QGIS software. Compared to the use of single data sources, the map fusion technique provides more information with more fitting results when producing thematic maps^44^. The final maps are based on the fusion of a set of different validated data provided by official agencies and institutions (Table 2), as described below:

- the ESA World Cover 10 m resolution LULC open and free product^45^, which presents an overall accuracy of over 75% (https://esa-worldcover.org/en);
- the National Crop Map developed by the National Institute of Agricultural Technology (INTA), which is an Argentinian periodic crop cover map with an overall accuracy of over 90% (http://www.geointa.inta.gob.ar/);
- the map of horticultural crops from the peri-urban area of Córdoba city generated for the year 2019, which was elaborated by the Gulich Institute—National Commission of Spatial Activities (IG-CONAE), which is an important input in the frame of river irrigation uses for Córdoba province (https://geocatalogos.conae.gov.ar).

**Table 2:** Description of LULC datasets.

Source	Data base	Period	Spatial resolution
ESA World Cover	Sentinel-1 & Sentinel-2	2019	10 m/px
National Crop Map	Landsat 8	2019–2020 2 Seasons	30 m/px resampled to 10 m/px
Land Cover and Land Use Map of Córdoba City Periurban Area	Sentinel-2	2019	10 m/px

The thematic maps were generated through machine learning based on a methodological and algorithmic decision tree. This technique is highly effective in satellite data analysis due to its ability to process large amounts of information and detect complex patterns^46^, and LULC time-series mapping has a great potential to analyze satellite data^47,48^. The final LULC thematic maps include seven classes: forest, shrubland, grassland, cropland, built-up, bare soil (including sparse vegetation), and permanent water bodies. Furthermore, the cropland class was reclassified using the maps from INTA and IG-CONAE to highlight the diversity of sub-type seasonal crops (corn, soy, alfalfa, fallow, winter cereals, and horticulture).

#### Buffer generation for multiscale approach

Two different buffers and spatial scales were selected. The local-scale analysis used a circular buffer area of 1000 m from each sample site to identify the most relevant surroundings LULC potentially affecting water quality. On the other hand, the macro-scale analysis to evaluate the relationship between water quality and LULC at a broader scale was carried out by defining a buffer area of 20,000 m length upstream and 5000 m width from each sample site. Intermediate scales that did not show significant results were excluded from the analysis.

### Statistical analysis

To examine the association between LULC classes and the water quality of the river as well as to identify possible pollution point sources by type of pollutant, a descriptive statistical analysis was performed on the water quality dataset. To assess significant differences between sample sites, an ANOVA analysis was performed.

The Pearson Correlation Coefficient (r) was calculated between physicochemical variables (conductivity, dissolved oxygen, turbidity, nitrite, nitrate, ammonium, hardness, chloride, sulfate, total coliform bacteria, temperature, pH), the WQIs, and LULC data at the two spatial scales and for the seasons (summer and winter). Principal Component Analysis (PCA) was applied to the dataset to identify the most representative water quality parameters that better explain the sources of variance^43,44^. Cluster analysis (CA) technique was applied to group the sampling sites according to the spatial variability of the physicochemical characteristics of water quality surveyed into the field^49^. The WQI and the correlation statistical analyses were carried out using R package, while the statistical descriptive analysis, the PCA, and the biplot were performed with Infostat software.

## Results

### Water quality descriptive analysis

**Figure 2:**
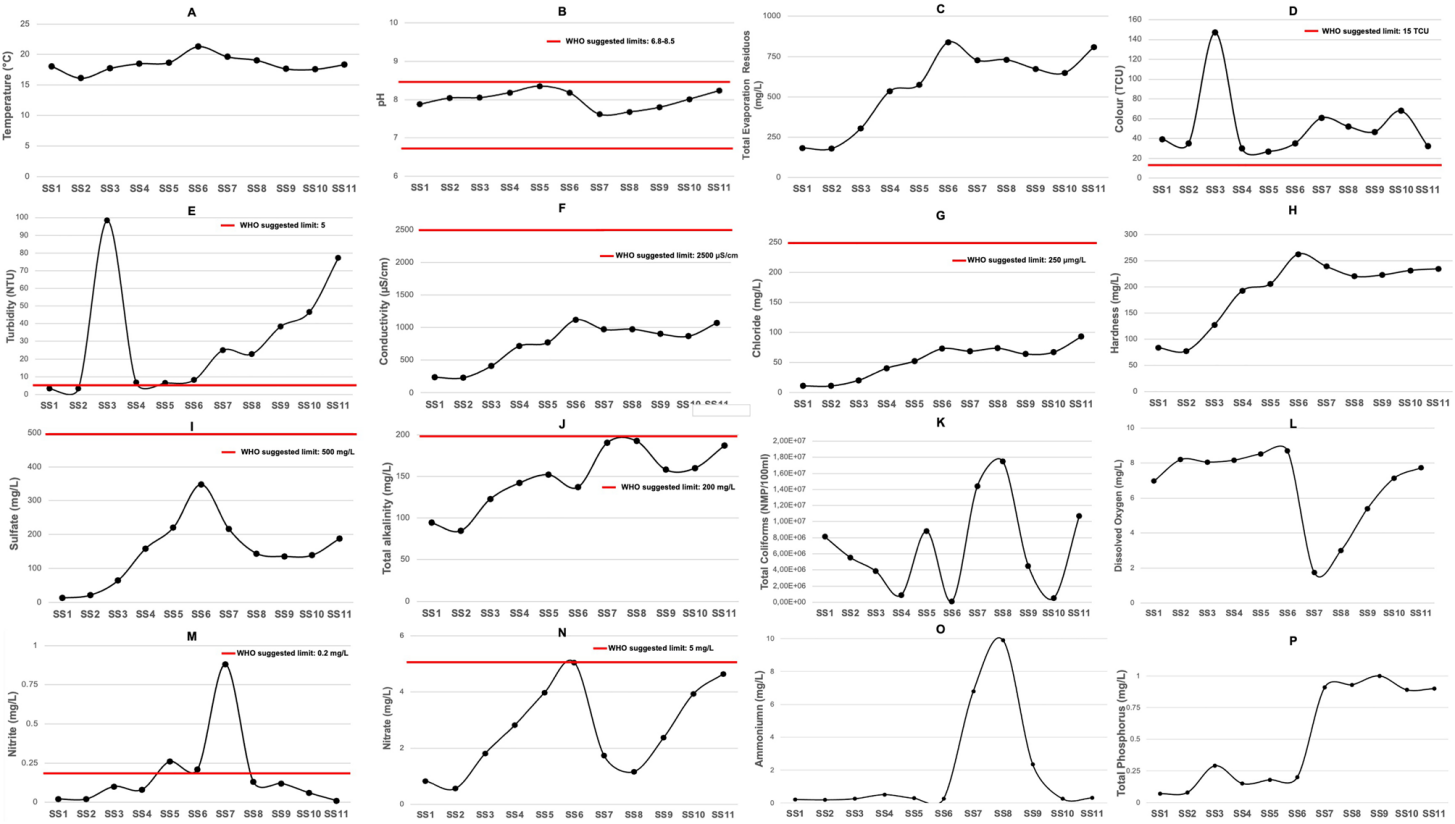
Sampling sites average measured variables per kilometer from San Roque Dam to Mar Chiquita Lagoon. (**A**) Temperature profile; (**B**) pH profile; (**C**) Total evaporation residues profile; (**D**) Color profile; (**E**) Turbidity profile; (**F**) Conductivity profile; (**G**) Chloride profile; (**H**) Hardness profile; (**I**) Sulfate profile; (**J**) Total alkalinity profile; (**K**) Total coliform profile; (**L**) Dissolved oxygen profile; (**M**) Nitrite profile; (**N**) Nitrate profile; (**O**) Ammonium profile and (**P**) Total phosphorus profile (for details Table S1).

The analysis findings indicated statistically significant differences between sample sites (*p* < 0.05) for most water quality variables, except for temperature, color, turbidity, and total coliforms. The data sets for all the water quality variables measured per sample sites along with World Health Organization (WHO) Guidelines are provided in Fig. 2. All sample sites were within acceptable limits for temperature, pH, chlorine, total evaporated residues, conductivity, chloride, hardness, sulfate, dissolved oxygen, ammonium, and total phosphorus. In the case of turbidity all sample sites, except SS1 and SS2, were over the suggested limited values. Finally, some values of nitrite (SS5, SS6, and SS7), nitrate (SS6), and total alkalinity (SS7 and SS8) at the sampling site level were above or outside the recommended limits. In addition, for the variables color and total coliforms, all sampling points exceeded the WHO-established limit values.

In addition, the behavior of the variables dissolved oxygen, ammonium, and nitrate- in the river profile is noteworthy. In the case of dissolved oxygen, Fig. 2 shows values near saturation concentration in atmospheric conditions and well-mixed water column, 8 mg/L, and drop suddenly at SS7 where the sewage discharge plant is located, reaching a value of 2 mg/L, which is much below from aquatic life guidelines values (4 mg/L)^50^. Consequently, ammonium, the most reduced form of nitrogen compounds, presents a high rise at SS7 which coincides with the anoxic conditions outlined before (Fig. 2). After that, it goes down due to oxidation processes as the river recovers dissolved oxygen in the water column. For aquatic life national regulations, the safe concentration limits of this compound are between 0.05 and 0.47 mg/L for the whole possible range of pH and temperature that can be found in natural surface waters^51^. The values found in this river are over that limit from the sewage discharge plant until 75 km from it. Ammonium can be discharged from wastewater, industry, and domestic waste among other sources^47^.

Figure 2 shows the nitrite concentration profile which starts to rise at point four when the river enters the downtown city and reaches its highest value at SS7 where the sewage discharge is located showing values greater than 0.8 mg/L, much higher than the WHO limit suggested (0.2 mg/L). This is consistent with reduced compounds that appear in anaerobic conditions when a huge amount of organic matter is being discharged as was explained in the ammonium case^52^. Nitrite is an intermediate compound in the oxidation of ammonia nitrogen to nitrate in water. It also can be a product of denitrifying bacteria in anaerobic sediment or water. Nitrite is ultimately oxidized to nitrate in the presence of dissolved oxygen. As can be seen in this figure, its concentration goes down once the river recuperates dissolved oxygen saturation values since it presents a short lifetime in oxygenated natural waters.

Figure 2 also presents the nitrate profile which rises while passing through Córdoba city, Sample Sites 3 to 7, then decreases while passing through rural areas and increases again in the track of agricultural lands, which coincides with nutrient discharges due to livestock and fertilization on crops lands^53^.

### Water Quality Index, seasonal and spatial behavior

**Figure 3:**
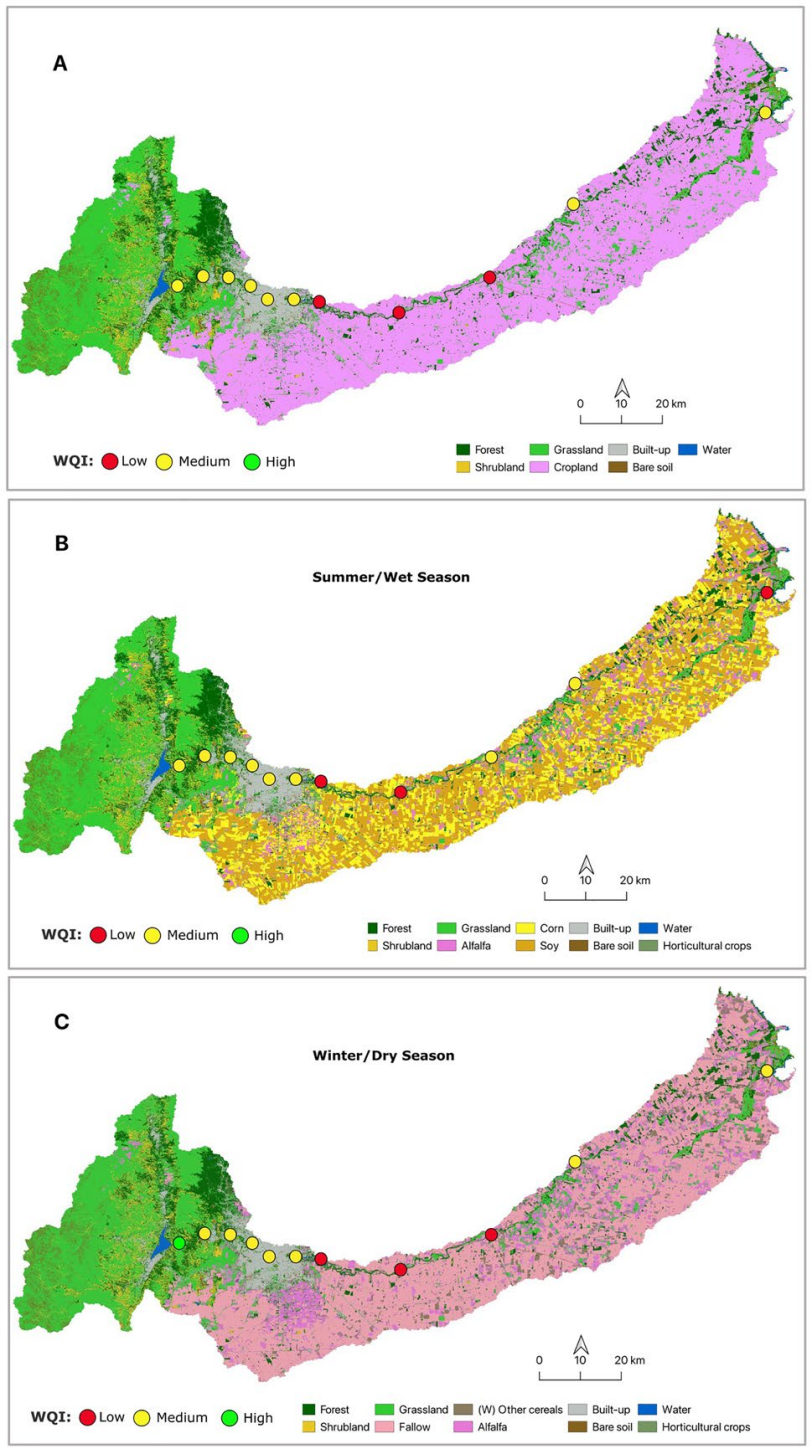
Mean and Seasonal Water Quality Index per Sample Site among LULC maps for the Suquía River Basin. (**A**) Mean Water Quality Index across ESA LULC map. (**B**) Summer Water Quality Index, overlaid on the ESA map with cropland class reinterpreted for summer crops. (**C**) Winter Water Quality Index, overlaid on the ESA map with cropland class reinterpreted for winter crops.

Figure 3 presents the spatial distribution of the WQIs. As expected, the results showed that sample sites 1 and 2 had the highest WQI values, which represented the best water quality condition. From sample SS3, as the river flowed through Córdoba City, the WQI decreased, with the worst WQI close to the wastewater treatment plant (SS7). From SS8, the WQI increases but does not reach the original values. In addition, results showed that, except for sample sites 7, 9, and 10, the cold season had a better WQI than the warm season.

### Satellite LULC products

The LULC map based on ESA classification has included 7 classes: forest, shrublands, grasslands, croplands, built-up areas, bare soil including sparse vegetation, and water (Fig. 3A). The Summer LULC thematic map has 12 classes (Fig. 3B): forest, shrublands, grasslands, croplands, herbaceous cover, corn, soy, alfalfa, sheltered horticultural crops, built-up areas, bare soil including sparse vegetation, and water. The Winter LULC thematic map has 11 classes (Fig. 3C): forest, shrublands, grasslands, croplands, herbaceous cover, fallow, others winter cereals, sheltered horticultural crops, built-up areas, bare soil including sparse vegetation, and water. It is evident that cropland is the most frequent land cover (about 54% of pixels), while the main summer crops are corn and soy, accounting for 19% and 26% of the total cropland pixels, respectively (Table 3); in winter, land is left mainly fallow (about 40% of cropland pixels) and to a lesser extent planted with some cereals (Table 3). Natural vegetation is mainly characterized by the presence of natural pastures and spontaneous herbaceous vegetation, with forest and scrub to a lesser extent (Table 3). Grasslands and forest, mainly located in the upper region of the basin, constituted the second and third-most dominant LULC classes, accounting for 24.44% and 9.63%, respectively. Shrubland represents approximately 4.26% of the LULC map in both seasons. Bare soil and sparse vegetation represented 2.43% of the total area of the basin. The built-up area represents more than 4% of the basin's coverage and is mostly represented by the urban area of Córdoba City (Table 3). Finally, permanent water bodies (notably the Suquía River and San Roque Reservoir) accounted for 0.38%.

**Table 3:** Land Use Land Cover classes (in % of pixels) in the Suquía River Basin.

Classes	LULC general (%)	LULC subclass (%)	LULC winter (%)	LULC summer (%)
Forest	9.63		9.63	9.63
Shrubland	4.26		4.26	4.26
Grassland	24.44		24.44	24.44
Bare soil—sparse vegetation	2.43		2.43	2.43
Built-up	4.34		4.34	4.34
Water	0.8		0.38	0.38
Cropland	54.52	Not classified crops	12.91	8.99
		Herbaceous covers	0.06	0.05
		Horticultural Crops	0.09	0.09
		Fallow	38.34	n/d
		Soy	n/d	26.08
		Corn	n/d	19.18
		Others winter cereals	2.97	n/d
		Alfalfa	0.11	0.08

n/d, No data.

### Buffer size selection: local and regional scales

The results indicate variability in water quality along the riverscape; these changes can be associated with LULC transitions. Table 4 presents the correlation (r-values) between water quality variables and the different LULC classes at the local scale, while Table 5 presents the r-values obtained at the regional scale. To discuss the main results, the strong relation recognized for each class was described at both scales.

**Table 4:** Pearson’s correlation matrix of Land Uses/ Land Covers.

Variable	LULC						
	Forest	Shrubland	Grassland	Bare soil	Cropland	Build-up	Water
WQI	0.083	0.459	− 0.325	− 0.190	− 0.677*	0.508	− 0.585
Nitrite	0.053	− 0.165	0.065	0.298	− 0.026	− 0.114	0.907**
Nitrate	− 0.561	− 0.600	0.150	− 0.256	0.016	0.361	− 0.245
Ammonium	0.043	− 0.166	0.284	0.070	0.604*	− 0.466	0.461
Conductivity	− 0.495	− 0.738**	0.439	− 0.192	0.558	− 0.079	0.147
Turbidity	0.048	− 0.333	0.604*	0.508	0.312	− 0.341	− 0.071
Temperature	− 0.233	− 0.299	− 0.206	− 0.031	− 0.034	0.209	0.225
pH	− 0.464	− 0.211	− 0.353	− 0.428	− 0.500	0.708*	− 0.560
Sulfate	− 0.579	− 0.638*	− 0.027	− 0.240	0.059	0.357	0.144
Chloride	− 0.390	− 0.679*	0.586	− 0.197	0.663*	− 0.234	0.125
Hardness	− 0.553	− 0.776**	0.423	− 0.142	0.492	− 0.008	0.176
Diss oxygen	− 0.268	0.039	0.039	− 0.290	− 0.594	0.667*	− 0.686
Total coliforms	− 0.302	0.015	− 0.455	− 0.356	− 0.404	0.538	− 0.147

Water Quality Index, and water quality variables at the local scale, showing correlation coefficient (r-values, measured from − 1 to 1) with *p* values.

Significant values are in [bold].

*p < 0.05; **p < 0.01.

**Table 5:** Pearson’s correlation matrix of Land Uses/Land Covers.

Variable	LULC						
	Forest	Shrubland	Grassland	Bare soil	Cropland	Build-up	Water
WQI	0.860**	0.788**	0.766**	0.470	− 0.599*	− 0.039	0.543
Nitrite	− 0.409	− 0.331	− 0.391	− 0.196	− 0.155	0.570	0.241
Nitrate	− 0.509	− 0.631*	− 0.465	− 0.432	0.146	0.359	− 0.592
Ammonium	− 0.481	− 0.385	− 0.558	− 0.177	0.449	− 0.092	− 0.248
Conductivity	− 0.918**	0.788**	− 0.902**	− 0.537	0.562	0.219	− 0.781**
Turbidity	− 0.073	− 0.253	− 0.219	− 0.280	0.365	− 0.261	− 0.409
Temperature	− 0.521	− 0.523	− 0.585	− 0.573	− 0.082	0.654*	0.419
pH	0.253	0.064	0.118	0.057	− 0.358	0.329	− 0.132
Sulfate	− 0.702*	− 0.742**	− 0.722*	− 0.434	0.066	0.649*	− 0.662*
Chloride	− 0.907**	− 0.930**	− 0.862**	− 0.491	0.682*	0.039	− 0.716*
Hardness	− 0.926**	− 0.968**	− 0.882**	− 0.579	0.506	0.291	− 0.817**
Diss. oxygen	0.491	0.351	0.437	0.214	− 0.442	0.145	0.137
Total coliforms	0.236	0.259	0.185	0.465	− 0.394	− 0.170	0.032

Water Quality Index, and water quality variables at the regional scale, showing correlation coefficient (r-values, measured from − 1 to 1) with *p* values.

Significant values are in [bold].

*p < 0.05; **p < 0.01.

No significant relationships (*p* > 0.05) were observed between the presence of bare soil (including sparse vegetation) and the variables considered in this study. Grasslands, at local scale, were significantly correlated with turbidity (r = 0.60, *p* < 0.05). While, at the regional scale the correlations were significant (*p* < 0.05) with WQI (r = 0.76), conductivity (r =  − 0.90), chloride (r =  − 0.86), hardness (r =  − 0.88), and sulfate (r =  − 0.72). At the local scale, the forest class was not significantly correlated with any considered variable. While at the regional scale, this land cover was significantly correlated with WQI (r = 0.86), conductivity (r =  − 0.91), sulfate (r =  − 0.70), chloride (r =  − 0.90), and hardness (r =  − 0.92). At the local scale, shrubland class was significantly correlated with conductivity (r =  − 0.73), sulfate (r =  − 0.63), chloride (r =  − 0.67), and hardness (r =  − 0.77). At the regional scale, this class was significantly correlated (*p* < 0.01) with WQI (r = 0.78), conductivity (r = 0.78), sulfate (r =  − 0.74) chloride (r =  − 0.93), and hardness (r =  − 0.96). These natural vegetation covers were strongly related to inorganic water quality variables and the results indicated a strong relationship between these cover classes and good water quality.

Build-up class was correlated (*p* < 0.05) with pH (r = 0.78) and dissolved oxygen (r =  − 0.66). Particularly for Suquía River, the pH values remained alkaline throughout all SSs, with a peak after passing through the urban area (SS 3) and a minimum after the water treatment plant discharge (SS 7). At the regional scale, the build-up class was correlated with temperature and sulfates (r = 0.65 and r = 0.64, respectively).

Water class was strongly correlated with nitrite at the local scale (r = 0.90, *p* < 0.01), which can be associated with the presence of lagoons, generated by arid extraction mining activity, which are located in areas with high social vulnerability and surrounded by open landfills that produce reduced chemical compounds due to the oxidation of high amounts of organic matter. At the regional scale water class showed a similar behavior as natural vegetation covers, being strongly correlated with conductivity (r =  − 0.78), sulfate (r =  − 0.66) chloride (r =  − 0.71), and hardness (r =  − 0.81).

The analysis of cropland class revealed a significant (*p* < 0.05) negative relationship with the WQI at the local and regional scales, r =  − 0.67 and r =  − 0.59 respectively. Furthermore, the presence of agricultural land covers exhibited positive correlations with chloride at both scales and with ammonium specifically at the local scale. For a better understanding of the relationship between this land use and water quality, Table 6 shows the r-values obtained for each crop for each climatic season and water quality at both local and regional scales, respectively.

**Table 6:** Pearson’s correlation matrix of seasonal crops, Water Quality Index, and water quality variables at the local and regional scales, showing correlation coefficient (r-values, measured from − 1 to 1) with *p* values.

Variable	Local scale				Regional scale			
	Summer crops		Winter crops		Summer crops		Winter crops	
	Corn	Soy	Fallow	Alfalfa	Corn	Soy	Fallow	Alfalfa
WQI	− 0.396	− 0.396	− 0.018	− 0.640*	− 0.625*	− 0.49**6**	− 0.496	0.160
Nitrite	0.127	− 0.105	− 0.210	0.948**	− 0.127	− 0.170	− 0.158	0.805**
Nitrate	− 0.583	− 0.248	− 0.508	− 0.189	0.109	0.064	0.024	− 0.278
Ammonium	0.416	0.781**	0.585	0.488	0.559	0.468	0.549	0.811**
Conductivity	− 0.235	0.344	− 0.065	0.227	0.572	0.513	0.517	0.020
Turbidity	− 0.252	0.033	− 0.179	− 0.058	0.350	0.314	0.291	− 0.118
Temperature	− 0.360	0.091	− 0.264	0.303	− 0.001	− 0.105	− 0.064	0.468
pH	− 0.384	− 0.496	− 0.396	− 0.542	− 0.352	− 0.406	− 0.429	− 0.551
Sulfate	− 0.317	− 0.010	− 0.288	0.228	0.088	0.024	0.028	0.293
Chloride	− 0.172	0.388	− 0.003	0.198	0.700*	0.618*	0.621*	0.226
Hardness	− 0.315	0.251	− 0.168	0.249	0.490	0.453	0.444	0.296
Diss oxygen	− 0.346	− 0.562	− 0.352	− 0.701*	− 0.484	− 0.459	− 0.500	− 0.753**
Total coliforms	0.429	0.610*	0.489	0.441	0.487	0.326	0.413	0.616*

Significant values are in [bold].

*Significant, with *p* less than 0.05; **Significant, with *p* less than 0.01.

For summer crops, at regional scale corn showed significant correlations (*p* < 0.05) with chloride (r = 0.70) and WQI (r =  − 0.62). At the local scale, soy was strongly correlated (*p* < 0.05) with ammonium (r = 0.78) and total coliforms (r = 0.61) while at the regional scale, soy showed strong correlations with chloride (r = 0.61). In the case of winter crops, fallow only showed strong correlations regional scale with chloride (r = 0.61). At the local scale, alfalfa was correlated with WQI (r =  − 0.64), nitrite (r = 0.94), and dissolved oxygen (r = 0.70). While at the regional scale, alfalfa crops were strongly correlated (*p* < 0.01) with nitrite (r = 0.80), ammonium (r = 0.81), dissolved oxygen (r =  − 0.75), and total coliforms (r = 0.61).

### Principal component analysis

**Figure 4:**
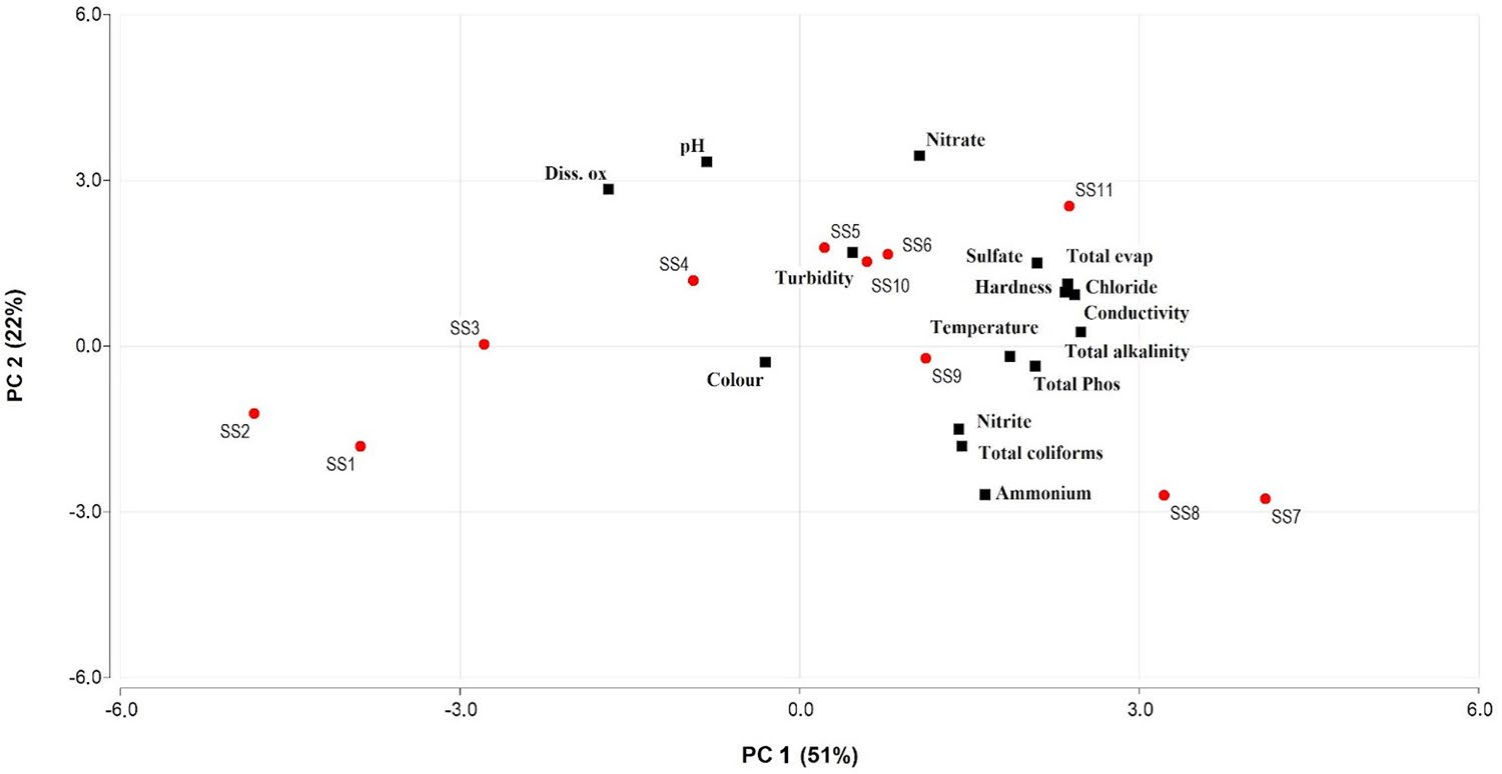
The PCA analysis of the complete data set with each of the sampling points highlighted in red and with the number. This graphic represents the two main dimensions of the PCA that contributed to explain 51% (PC1) and 22% (PC2) of the variation in the dataset (for details Table S2).

**Figure 5:**
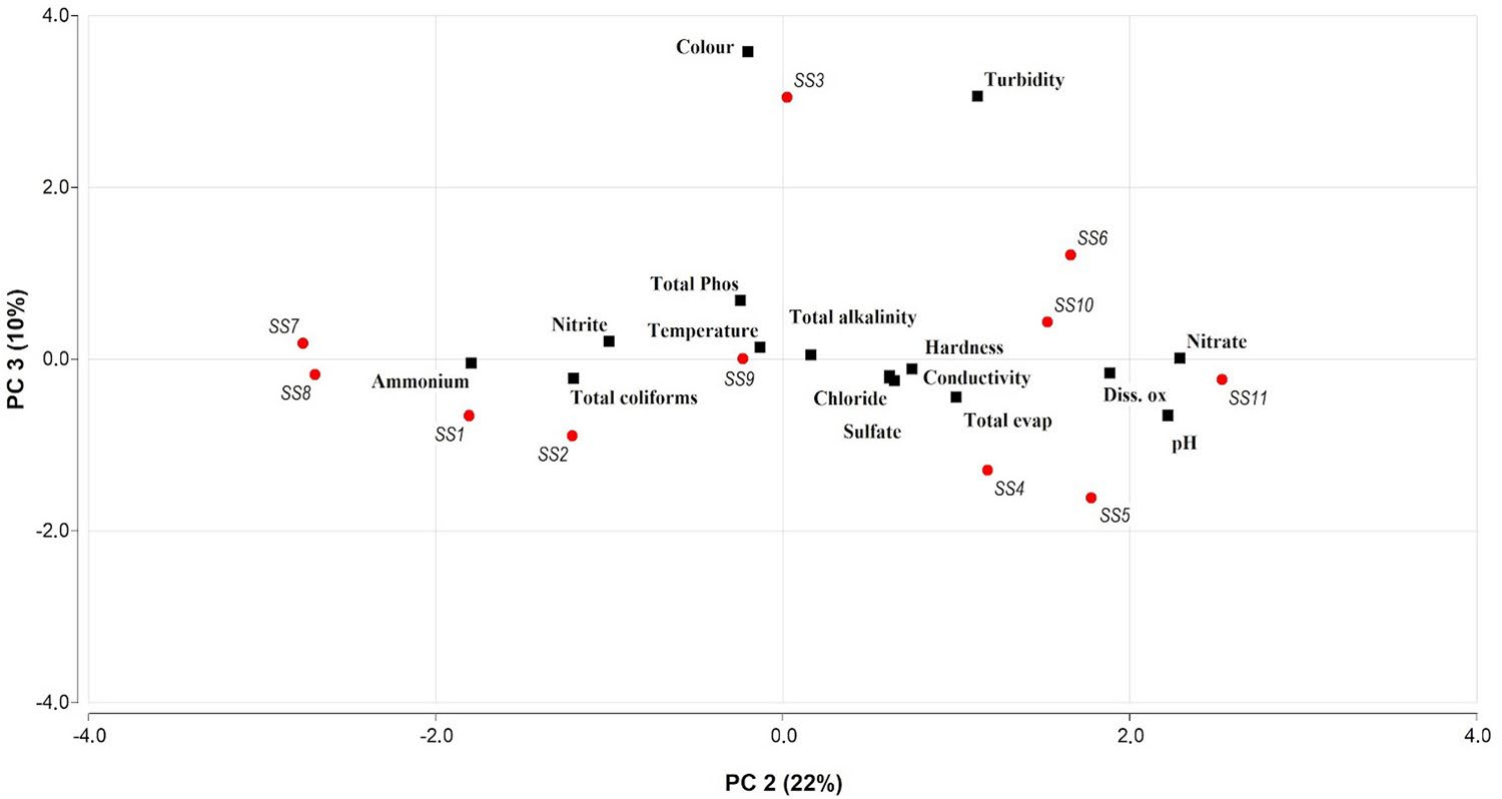
The PCA analysis of the complete data set with each of the sampling points highlighted in red and with the number. This graphic represents the second and the third dimension of the PCA that contributed to explain 22% (PC2) and 10% (PC3) of the variation in the dataset (for details Table S2).

Three PCs with eigenvalues equal to or greater than 1.0 were retained and used to understand the data structure^54,55^ and to identify possible pollutant sources^56^. These three PCs explained 83% of the total variance in the water quality dataset contributing 51% (PC1), 22% (PC2), and 10% (PC3). The first component (PC1) is related to the presence of solutes of inorganic origin, mainly conductivity (0.33), chlorides (0.32), hardness (0.33), total evaporation residue (0.33), and alkalinity (0.34), these components could be associated with the inorganic variables. The second component (PC2) involved pH (0.46), dissolved oxygen (0.39), ammonium (− 0.37), and nitrate (0.47), components that can be related to the organic aspects and are associated mainly with sewage discharges. The third component (PC3) mainly involves color (0.74) and turbidity (0.63) which are components associated with agricultural erosion and with pollution from domestic wastewater discharges and open illegal landfills at the riverside. Figure 4 shows the graphic of the two main dimensions of the PCA (PC1 and PC2) representing 73% of the variation in the dataset, while Fig. 5 shows the graphic for PC2 and PC3, representing 32% of the variation in the dataset.

The PCA analysis reveals that the greatest variability in the data on river water quality is associated with inorganic variables that are linked to the accumulation of dissolved solids entering the river through runoff. Previous studies in this basin showed that the sampling sites can be grouped into five clusters^36^. The first one was associated with the sewage effluent treatment plant (SS7), and the second site (SS8) after the plant, was characterized by an area of mixed land use. The third cluster, in the lower basin, was characterized by agricultural LULC mainly (SS9, SS10, and SS11); the fourth group was associated with urban LULC (SS4, SS5 and SS6); and the last group (SS1, SS2 and SS3), in the upper basin, was associated principally with non-anthropic land uses, but additionally, organic pollutants contribute to water contamination in Suquía River on those SS as can be seeing in Fig. 2.

### Cluster analysis

**Figure 6:**
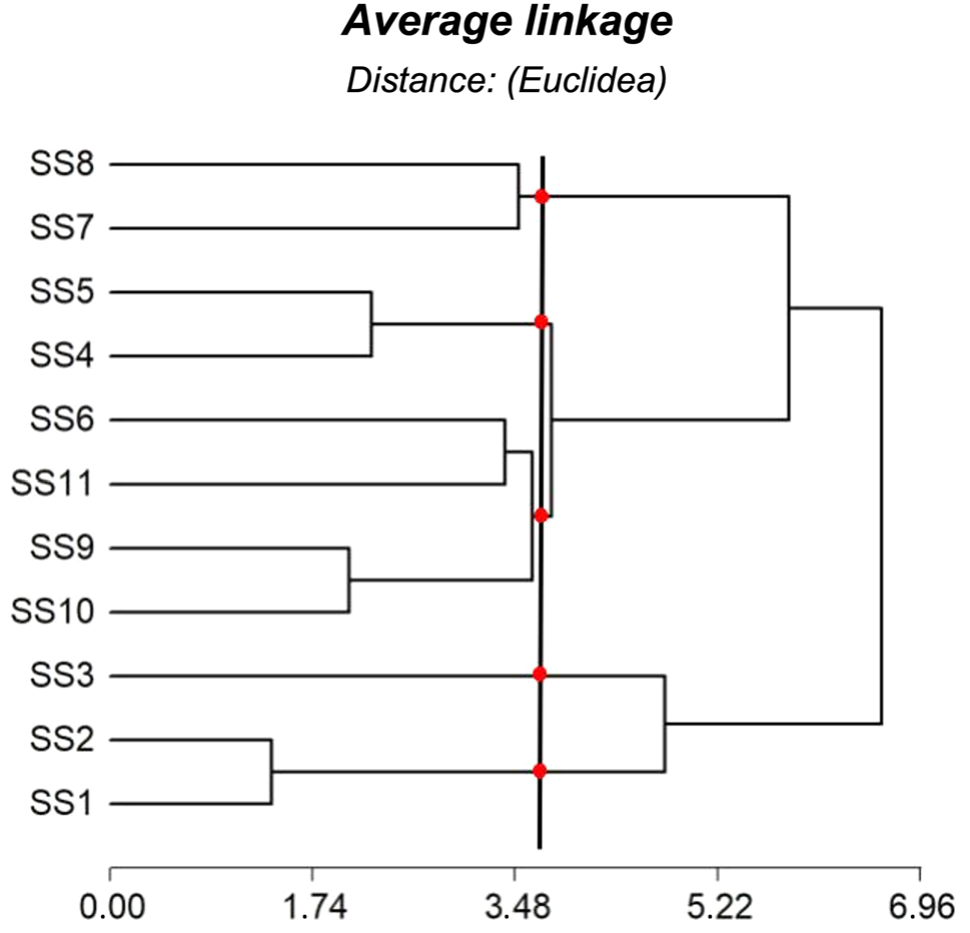
The cluster analysis of the complete data set with each of the selected clusters highlighted with a red point.

The resulting cluster classification (Fig. 6) has ordered the sampling sites into 5 groups according to their internal homogeneity-of the cluster- and external heterogeneity (between clusters). Cluster 1 represents sampling sites 1 and 2 in the upper basin, and was associated principally with non-anthropic land uses. The second cluster corresponds to Sample Site 3, also in the upper basin, where organic pollutants contribute to water contamination in Suquía River as can be seen in Fig. 2. Cluster 3 grouped the sampling sites number 6, 9, 10 and 11, characterized mainly by agricultural LULC. Cluster 4 grouped sample sites 4 and 5 which are mainly associated with urban LULC. Finally cluster 5 includes sample sites number 7 and 8 that can be associated with the sewage effluent treatment plant, and refers to an area characterized by mixed land use. Previous preliminary studies in this basin showed similar results^36^.

## Discussion

The research highlights findings regarding the variables of color and total coliforms, indicating that all sampling points surpassed the limit values set by the WHO. Particularly, the Suquía River exhibited high levels of fecal contamination, with coliform bacteria counts significantly exceeding acceptable limits for the microbiological quality of recreational waters for all SS. Amé et al. ^33^ suggested that the elevated values of these variables beyond WHO-recommended limits could be attributed to the impact of Córdoba City on the river's water quality. Pesce and Wunderlin^34^ also found total coliforms along SS located in Córdoba City and near to the Bajo Grande treatment plant, indicating a deterioration of Suquía River water quality mainly determined from sewage sources. Furthermore, the presence of virus contamination in the Suquía River was documented^32^. Although there was no observed association between bacterial indicators and viral contamination in the Suquía River, it is essential to note that recreational waters in this area are not subjected to any treatment, which is a potential hazard to public health. This situation also may necessitate an increase in the costs associated with treating the river water to make it suitable for drinking purposes.

The seasonality could affect the results related to the water quality and, except for sample sites 7, 9, and 10, the cold season had a better WQI than the warm season. These results are in disagreement with different authors who suggest that during the wet season (local summer), rainfall could cause better surface water quality since it naturally dilutes the pollutants, thus decreasing pollutant concentrations and improving water quality^48,49^. Particularly, during the Summer, WQI at SS11 drops concerning SS10. This could be associated with a higher percentage of soy croplands in this region in summer, which correlates positively with ammonium concentration in water, see Table 6, which can be produced from fertilizers used in nearshore cropped soils^57^. In addition, 2018 was a dry year in the basin and dilution of pollutants was not so efficient as other years^58,59^.

The influence of different buffer sizes is an important aspect to be considered^24,60,61^. In this study, the relationship between water quality and LULC patterns in the Suquía River was assessed at local and regional buffer scales. Each land use class was examined separately to understand its impact on water quality at both scales. According to Shi et al.^62^, there is currently no consensus among authors who have employed this methodology regarding the optimal buffer size for effectively interpreting the relationship between water quality and the surrounding land use and land cover at sampling sites, mainly because some environmental assessments are scale-dependent in space and time. This is in accordance with several authors, who concluded that local and regional scales have proven to be the most influential in determining the effectiveness of buffer zones as descriptive^63,64^. Heidkamp and Christian^65^ studied water quality in the Charles River watershed by calculating the percent ground cover at the subbasin and 100-m local buffer zone scale and found that macroinvertebrates inhabiting that ecosystem were more sensitive to development within the buffer zone than at the sub-basin scale. Gove et al.^25^ found that LULC within the basin plays a critical role in controlling the quantity, form, and timing of various material inputs affecting water quality. Since intermediate scales were taken into account, but their results were not as significant and therefore not included, this study also highlights the influence of different buffer sizes as an important aspect to be taken into account in this type of research. Ultimately, the selection of an appropriate buffer size will depend on the specific objectives and nature of each study case.

For what concerns the effect of LULC on water quality, the results indicate variability in water quality along the riverscape; these changes can be associated with LULC transitions. The natural LULC vegetation covers were strongly related to inorganic water quality variables and the results indicated a strong relationship between these cover classes and good water quality. In this sense, Norris^66^ has already indicated the importance of local stream-adjacent vegetated buffer zones, which would be determined not only by their capacity to regulate water quality but also by a series of additional benefits linked to the provision of ecosystem services. Furthermore, Lee et al.^67^ investigating the spatial configuration of land uses in relation to water quality by using landscape metrics, found that the widespread distribution of forests could improve the water quality of the watershed. This is followed by Chua et al.^53^, who highlighted the advantages of maintaining the riparian vegetation conditions to improve the quality of the water resource.

The anthropic build-up class has shown its effect in terms of temperature. Although no statistically significant differences were found between temperature and SS, the temperature profile for SS along the river showed a gradual increase from the entrance to Córdoba City (SS 3) to the city exit (SS 6), which can be attributed to the river's channelization with concrete material. According to Chen et al.^68^, river bends play an important role in the health and productivity of the riverine ecological system and are shaped by human intervention. Additionally, the increase in temperature can be also influenced by the surface heat island effect in Córdoba City^69^ where the temperature of the average summer day can be 4 °C higher than that in its surrounding areas. The analysis of cropland class revealed a negative relationship with the WQI at the local and regional scales, since the presence of agricultural land covers exhibited positive correlations with chloride at both scales and with ammonium specifically at the local scale. Croplands have also highlighted the importance of seasonal assessment of water quality, since they are different in summer and in winter. As in other case studies^11,70^, croplands have resulted in the most common LULC of the area (54.52%) and it is evident how the unsustainable use of chemical (inorganic and organic) inputs can strongly affect river water quality. These fertilizers can be the primary cause of non-point source of water pollution, and the seasonality with dry or wet season can mitigate or increase, respectively, the chemical pressure on water quality.

For summer crops, at regional scale soy and corn crops showed significant correlations with chloride, while, at local scale, corn were strongly correlated with ammonium and total coliforms. In this sense, Mwangangi et al.^71^ indicated that high chloride levels can reduce water quality in a runoff, especially upstream. Oberhelman and Peterson^72^ found that potassium chloride fertilizers can contribute to raising stream baseflow chloride concentrations; and also reveal the potential for agricultural sources of chloride to contribute to salinization in rural and urban–rural watersheds.

In the case of winter crops different significant correlations were identified, however the relationship between agricultural intensification and the increases in nitrate compounds affecting water quality has been observed by different researchers^73–75^. Salon et al.^76^ consider that the nitrogen acquisition on legume covers may be spatially and temporally limited, while Thapa et al.^77^ suggest that non-leguminous cover crops can represent an effective way to reduce nitrate leaching. Hatfield et al.^78^ in a study conducted in the Raccoon River watershed in the United States found that nitrate–N loads have decreased slightly due to increased crop yields and increased nitrogen removal in corn and soy; while they described a significant correlation between watershed acreage devoted to small grains and hay and increased nitrite due to altered seasonal water use patterns and nitrogen loss during fall or early spring water movement, in contrast to corn or soy, which have a limited pattern of concentrated nitrogen uptake between June and early September in these this region. Brumberg et al.^79^ demonstrated that increasing agriculture activity in the riparian zone of streams consistently deteriorates water quality while advocating the effectiveness of riparian forest buffers in preserving river water quality.

This study has demonstrated quantitatively that, in Suquia's basin, surface water quality is negatively affected by agricultural and urban activities, while natural vegetated LULC classes had a positive impact, indicating the need to minimize the impact of human activities on water resources through sustainable land use practices. Further, this study has emphasized the importance of choosing appropriate buffer sizes when assessing LULC effects on water quality. At local-scale agricultural land use has led to elevated nitrite and ammonium levels, while chloride levels were high at regional scales. The research has underlined the significant correlation between water quality and seasonal crops, accentuating the importance of prudent seasonal crop selection and agricultural practices. These findings can be used as valuable guidance for decision-making and strategic management initiatives aimed at maintaining clean and available water resources. The different LULC can play an important role in maintaining the water quality also in consideration of the potential ecological role that they can play in providing different landscape services. A previous study focused on landscape changes in this area highlighted four spatial change indexes that show how the North-East and the South-East areas of the metropolitan area of Córdoba have faced important conversions from horticulture to extensive agriculture (soy, corn, etc.)^80^, with consequent effect on river water quality.

In general, future research must further investigate the long-term effects of sustainable land use practices on water quality and the overall ecological health of Suquia's basin. Additionally, it will be essential to explore the potential synergistic effects of combining different land use strategies and restoration approaches to maximize water resource protection and riverscape resilience. Furthermore, understanding the socio-economic implications of implementing these strategies and their acceptance by local communities will be crucial for successful and sustainable water resource management in the region.

## Conclusions

The spatialization of the results can help strongly in assessing the diffusion of point and non-point pollution in the riverscape. Point source due to wastewater discharge and water urban runoff as well as agricultural activities resulted the main anthropogenic drivers for water quality parameters in the study area. However, the assessment at different spatial scales is important as well: the border spatial scale can delineate the general trend but finer spatial scales can highlight different trends at local scale. Not all LULC classifications are good for analyzing the relationship between land-use classes and the water quality, since broad classification cannot be able to catch the local effects of LULC on WQI (see ESA classification). The correct temporal scale can also play a crucial role since it allows the identification of the season more characterized by pollution also in consideration of climate change. Moreover, the implementation of good agricultural practices, such as the responsible use of fertilizers and efficient irrigation, is necessary to preserve water quality in the region. Given the important role played by surface water (rivers, lakes,…), intended as a non-renewable resource, the knowledge gained from this research can play a crucial role in water resources management, which supports the provision of river ecosystem services essential for the well-being of ecosystems and local populations. Finally, this study underscores the importance of utilizing the WQI as a tool to monitor the effectiveness of pollution mitigation strategies.

### Supplementary Information

Supplementary Information.

## Data Availability

The datasets used and/or analysed during the current study available from the corresponding author on reasonable request.
