# Supplementary material for: The multiscale nexus among land use-land cover changes and water quality in the Suquía River Basin, a semi-arid region of Argentina
Source: Sci Rep. 2024 Feb 26;14:4670. doi: 10.1038/s41598-024-53604-0 (PMC10897139; doi:10.1038/s41598-024-53604-0)
Supplement: Supplementary file 1 — Supplementary Information. [file 41598_2024_53604_MOESM1_ESM.pdf]

Appendix A - Supplementary Information

The multiscale nexus among land use-land cover changes and water quality in the Suquía River Basin, a semi-arid region of Argentina

Sofía Paná[1,2]; M. Victoria Marinelli[2]; Matías Bonansea[1,3*]; Anabella Ferral[1,2]; Donatella Valente[4*]; Vera Camacho Valdez[5]; Irene Petrosillo[4]

[1]Consejo Nacional de Investigaciones Científicas y Técnicas (CONICET), Av. Cdad. de Valparaíso S/N, Córdoba, Argentina
[2]Instituto Gulich, Universidad Nacional de Córdoba-CONAE, Centro Espacial Teófilo Tabanera, Ruta 45 km 8, Falda del Cañete, 5187, Córdoba, Argentina
[3]Departamento de Estudios Básicos y Agropecuarios, Facultad de Agronomía y Veterinaria (FAyV), Universidad Nacional de Río Cuarto (UNRC), Argentina
[4]Laboratory of Landscape Ecology, Dept. of Biological and Environmental Sciences and Technologies, University of Salento, Italy
[5]CONAHCYT- Departamento de Conservación de la Biodiversidad, El Colegio de la Frontera Sur, San Cristóbal de las Casas, México

***First Corresponding Author to which the proofs should be sent**
Dr. Donatella Valente, PhD
Department of Biological and Environmental Sciences and Technologies, University of Salento, Prov.le Lecce-Monteroni, 73100 Lecce, Italy. Tel. +39 0832 298
E-mail: donatella.valente@unisalento.it
***Second Corresponding Author:**
Dr. Matías Bonansea
Consejo Nacional de Investigaciones Científicas y Técnicas (CONICET), Av. Cdad. de Valparaíso S/N, Córdoba, Argentina.
E-mail: mbonansea@ayv.unrc.edu.ar

Table S1 - Physicochemical water quality variables were measured along 11 SSs on the Suquía River. M (Mean) Sd (Standard deviation) R (Range). Different letters (A, B, C, D) indicate statistically significant differences based on the LSD Fisher test ($p<0.05$).

Parameters		SS 1	SS 2	S S3	SS 4	SS 5	SS 6	SS 7	SS 8	SS 9	SS 10	SS 11
**Temperature**	M - Sd	18.07±5.24	16.12±4.71	17.7±4.71	18.5±5.84	18.7±5.72	21.3±6.93	19.6±4.81	19.05±5.74	17.6±5.21	17.6±5.64	18.3±5.48
	R	13-26	12.2-24.2	12.8-24.9	13.2-26	13.6-26	16.4-26.2	14.6-27	14-30	10-25.7	10.1-26.9	12.4-28
**pH**	M - Sd	7.88±0.33^{B}	8.04±0.35^{C}	8.05±0.43^{C}	8.18±0.33^{C}	8.35±0.42^{D}	8.18±0.42^{C}	7.62±0.17^{A}	7.68±0.17^{A}	7.8±0.17^{B}	8.01±0.23^{C}	8.23±0.37^{D}
	R	7.43-8.38	7.69-8.55	7.5-8.89	7.81-8.75	8.01-9.1	7.88-8.48	7.4-7.85	7.33-7.82	7.52-8	7.51-8.18	7.44-8.51
**Dissolved oxygen**	M - Sd	6.97±1.8^{B}	8.2±1.29^{C}	8.06±1.53^{C}	8.17±1.8^{C}	8.53±1.32^{C}	8.7±0.71^{C}	1.74±1.99^{A}	2.99±1.91^{A}	5.4±1.07^{B}	7.14±0.69^{C}	7.73±0.89^{C}
	R	3.6-9.2	6.6-9.8	6-10.8	6.4-11.4	7.2-10.8	8.2-9.2	0-5.6	0.1-4.6	4.2-6.8	6.4-8.2	6.11-8.6
**Color**	M - Sd	39.26±30.34	35±17	147±306	30±20.7	26.7±14.7	35±28.3	60.71±31	52.1±28.8	46.7±38.8	67.9±107.	32.1±21.8
	R	5-95	25-65	10-840	5-60	10-50	15-55	20-105	5-95	25-125	10-310	10-65
**Conductivity**	M - Sd	239.3±77.4^{A}	231±34.1^{A}	411±192^{B}	720±402^{B}	771±447^{B}	1120±790^{C}	972±411^{C}	977±361^{C}	903±349^{C}	868±284^{C}	1071±303^{C}
	R	157-359	195-272	252-743	298-1273	359-1568	561-1679	508-1566	553-1446	516-1339	511-1188	548-1445
**Turbidity**	M - Sd	3.32±2.82	3.5±1.14	98.3±238	6.8±3.97	6.53±3.56	8.15±4.88	25.04±27.1	22.9±13.2	38.4±31.3	46.7±35.1	77.3±75.3
	R	1.04-8.7	2.39-5.28	3.43-640	2.96-11.8	2.54-11.5	4.7-11.6	8.65-86	5.2-41.5	1.72-93	9.75-97	34.8-246
**Total evaporation residue**	M - Sd	185±50.4^{A}	178±26.2^{A}	303±131^{B}	535±310^{B}	575±347^{B}	838±616^{C}	726.71±322^{C}	729±288^{C}	673±277^{C}	648±226^{C}	807±235^{C}
	R	131-257	150-209	194-532	229-966	257-1189	402-1274	365-1188	396-1097	369-1016	366-901	415-1096
**Total alkalinity**	M - Sd	94.6±43.5^{A}	84.8±16.7^{A}	122±39.4^{B}	142.3±50.6^{B}	152±56.2^{B}	161±77.8^{B}	190±68.5^{C}	192±68^{C}	1583±59.6^{B}	160±43.5^{B}	187.14±42.3^{C}
	R	60-160	58-98	80-188	94-206	98-228	106-216	110-296	120-280	96-252	100-216	128-236
**Chloride**	M - Sd	10.9±2.41^{A}	10.8±2.68^{A}	20.4±10.7^{A}	40.5±22.2^{B}	52.33±33.8^{B}	73±58^{B}	68.9±28^{C}	73.6±35.9^{D}	64±30.5^{C}	67.1±26.9^{C}	93.1±31^{D}
	R	8-15	8-15	10-37	22-73	24-108	32-114	29-110	40-130	34-116	31-100	48-138
**Hardness**	M - Sd	84±35.8^{A}	77.6±13.3^{A}	127.1±48.9^{B}	193±89.4^{B}	206±99.5^{B}	263±176^{B}	239±82.6^{C}	220±63.6^{C}	223±77.9^{C}	231±72.3^{C}	234±58.4^{C}
	R	58-140	60-94	82-196	108-328	114-356	138-388	124-344	158-336	136-344	140-312	150-300
**Sulfate**	M - Sd	13.24±5.58^{A}	22±11.2^{A}	64.4±61.1^{B}	158±148^{C}	220.5±195^{D}	347±367^{D}	216±179^{C}	114.2±90.7^{D}	135.6±79.5^{C}	138±75.0^{C}	188±60.9^{D}
	R	10-25.1	10-34.2	10-187	36.6-447	52.5-567	87.7-607	10-560	33.4-239	27.5-247	25.6-233	92.5-243
**Total phosphorus**	M - Sd	0.07±0.03^{A}	0.08±0.03^{A}	0.29±0.38^{A}	0.15±0.06^{A}	0.18±0.05^{A}	0.2±0^{A}	0.91±0.28^{B}	0.93±0.37^{B}	1±0.62^{B}	0.89±0.25^{B}	0.9±0.22^{B}
	R	0.02-0.12	0.05-0.13	0.06-1.15	0.06-0.22	0.14-0.28	0.2-0.2	0.46-1.3	0.48-1.32	0.4-2.19	0.46-1.15	0.57-1.11
**N-ammonium**	M - Sd	0.22±0.27^{A}	0.19±0.13^{A}	0.26±0.25^{A}	0.51±0.53^{A}	0.29±0.17^{A}	0.26±0.16^{A}	6.8±4.22^{B}	9.9±8.07^{B}	2.34±2.09^{A}	0.26±0.29^{A}	0.31±0.42^{A}
	R	0.05-0.79	0.07-0.34	0.06-0.75	0.1-1.43	0.11-0.57	0.14-0.37	2.27-13.8	2.57-24.2	0.25-5.77	0.05-0.83	0.05-1.23
**N-nitrite**	M - Sd	0.02±0.01^{A}	0.02±0.01^{A}	0.1±0.07^{A}	0.08±0.07^{A}	0.26±0.19^{A}	0.21±0.17^{A}	0.88±0.94^{B}	0.13±0.1^{A}	0.12±0.07^{A}	0.06±0.08^{A}	0.01±0^{A}
	R	0.01-0.04	0.01-0.02	0.02-0.18	0.01-0.21	0.04-0.46	0.09-0.33	0.01-2.7	0.01-0.28	0.01-0.2	0.01-0.23	0.01-0.01
**N-nitrate**	M - Sd	0.83±1.28^{A}	0.58±0.13^{A}	1.81±1.36^{B}	2.82±1.61^{C}	3.98±2.25^{C}	5.05±4.03^{C}	1.73±1.04^{B}	1.16±0.89^{B}	2.37±1.34^{B}	3.93±1.21^{C}	4.64±1.48^{D}
	R	0.2-3.7	0.4-0.7	0.3-4.2	1.1-5.1	1.5-8	2.2-7.9	0.03-3	0.2-2.4	0.9-4.1	2.4-5.9	2.3-6.3
**Total coliform bacteria**	M - Sd	8,12E+06±1,23E+07	5,55E+06±1,04E+07	3,85E+06±8,93E+06	8,88E+051,18E+06	8,81E+06±1,18E+07	1,22E+05±1,66E+05	1,44E+07±1,20E+07	1,75E+07±1,11E+07	4,50E+06±9,59E+06	5,16E+05±8,35E+05	1,07E+07±1,25E+07
	R	1,10E+04-2,40E+07	1,10E+04-2,40E+07	2,40E+04-2,40E+07	2,40E+04-2,40E+06	2,40E+04-2,40E+07	4,60E+03-2,40E+05	2,40E+05-2,40E+07	1,50E+03-2,40E+07	2,40E+04-2,40E+07	1,10E+04-2,40E+06	2,40E+04-2,40E+07

Table S2 - Principal Component Analysis (PCA) for the APRHI dataset.

**Variables**	**CP1**	**CP2**	**CP3**
Temperature	0.26	-0.03	0.03
pH	-0.11	**0.46**	-0.14
Dissolved Oxygen	-0.23	**0.39**	-0.03
Color	-0.04	-0.04	**0.74**
Conductivity	**0.33**	0.13	-0.04
Turbidity	0.06	0.23	**0.63**
Total evaporation residue	**0.33**	0.13	-0.05
Total Alkalinity	**0.34**	0.03	0.01
Chloride	**0.32**	0.13	-0.05
Hardness	**0.33**	0.15	-0.02
Sulfate	0.29	0.21	-0.09
Total phosphorus	0.29	-0.05	0.14
N-Ammonium	0.23	**-0.37**	-0.01
N-Nitrite	0.19	-0.21	0.04
N-Nitrate	0.15	**0.47**	-0.03
Total Coliform bacteria	0.20	-0.25	-0.05
% total variance	51	22	10
Cumulative % variance	51	73	83
Eigenvalue	8.21	3.58	1.66
